# Pesticide Contamination in Native North American Crops, Part II—Comparison of Flower, Honey Bee Workers, and Native Bee Residues in Lowbush Blueberry

**DOI:** 10.3390/insects15080567

**Published:** 2024-07-26

**Authors:** Francis A. Drummond, Anne L. Averill, Brian D. Eitzer

**Affiliations:** 1 School of Biology and Ecology, and Cooperative Extension, University of Maine, Orono, ME 04469, USA; 2 Department of Environmental Conservation, University of Massachusetts, Amherst, MA 01003, USA; averill@eco.umass.edu; 3 Department of Analytical Chemistry, The Connecticut Agricultural Experiment Station, New Haven, CT 06504, USA; brian.eitzer@ct.gov

**Keywords:** pesticide exposure, *Apis mellifera* (L.), *Bombus* spp., *Osmia* spp., flowers, pollen

## Abstract

**Simple Summary:**

Working in lowbush blueberry fields, we studied how to provide a holistic measure of pesticide exposure to bees. Several different taxa of bees can be involved in blueberry pollination, including honey bees, bumble bees (which are native), and other native bees, such as mason, mining, and leafcutter bees. Using chemical analysis, we compared and contrasted samples from different sources to (1) measure pesticide exposure and (2) predict exposure risk in light of established levels of concern established by the U.S. Environmental Protection Agency. We trapped honey bee pollen as foragers entered the hive and, within the three years of the study, found considerable similarity of these samples to treated flowers; the measures were the number and concentration of pesticide residues, as well as the predicted level of risk. We found differences among two or three of these measures when comparing trapped honey bee pollen and trapped mason bee pollen, as well as between trapped honey bee pollen and whole bodies of captured honey bees. In these comparisons, the number of residues detected in honey bee pollen was typically greater, as was the estimated risk. The residues found when comparing whole body analysis of honey bee workers, bumble bee queens, and other native bees showed similarity in total residue concentrations and risk, but there was a higher number of different residues detected on honey bees. This research complements another study (Part I) focused on honey bee pollen trapping in lowbush blueberry and cranberry. Both studies provide a toxicological baseline for pesticide residue exposure to pollinators in the lowbush blueberry agroecosystem.

**Abstract:**

In lowbush blueberry fields, we conducted residue analysis comparing flowers, trapped pollen (honey bee and *Osmia* spp.), and collected bees (honey bee workers, bumble bee queens, and non-*Bombus* spp. wild native bees). The study was conducted from 2012 to 2014. The number of pesticide residues, total concentrations, and risk to honey bees (Risk Quotient) on flowers were not significantly different from those determined for trapped honey bee pollen (except in one study year when residues detected in flower samples were significantly lower than residue numbers detected in trapped pollen). The compositions of residues were similar on flowers and trapped pollen. The number of residues detected in honey bee pollen was significantly greater than the number detected in *Osmia* spp. pollen, while the total concentration of residue was not different between the two types of pollen. The risk to honey bees was higher in trapped honey bee pollen than in trapped *Osmia* spp. pollen. The analysis of honey bee workers, native bumble bee queens, and native solitary bees showed that although more pesticide residues were detected on honey bee workers, there were no differences among the bee taxa in total residue concentrations or risk (as estimated in terms of risk to honey bees).

## 1. Introduction

Worldwide, bees are important pollinators of wildflowers and crop plants [1,2,3,4,5,6]. Native wild bees, as well as honey bees, are important pollinators of crops, although it is possible that there are many crop systems where wild bees contribute to significant fruit set despite an abundance of honey bees [7]. Bees in cropping systems appear to be at greater risk in agricultural landscapes than in natural habitats [7,8,9]. Pesticide exposure has been found to be one of the causes of bee decline [10,11,12]. The negative effects of pesticide exposure are not only due to direct acute mortality [13] but also to the chronic impacts resulting in a reduction in reproduction [14], increased susceptibility to disease [15], and repellency of bees to contaminated forage [16]. Agricultural intensification, including pesticide exposure to bees, has been shown to increase the risk to fruit set and crop yield [17,18]. Studies on the extent and degree of bee exposure to pesticides and their consequences have received much recent attention [19,20,21,22,23]. However, much more research needs to be conducted in order to determine various ways that pesticide exposure can vary in a single bee species, vary over multiple bee species, be mediated by landscape and management tactics, and how this knowledge can be used to minimize exposure to foraging bees. 

This study focused on pesticide exposure to bees in lowbush blueberry. Lowbush blueberry comprises five species of wild *Vaccinium*, dominated by the sweet lowbush blueberry, *Vaccinium angustifolium* Aiton [24]. This native fruit crop is an important economic crop in Maine, U.S.A., as well as Quebec, Canada, and the entire Canadian Maritime region. It is a mass flowering wild fruit crop that has not been genetically improved and is not planted. Lowbush blueberry fields are developed by the removal of trees in the forests, allowing the natural understory lowbush blueberry plants to grow vegetatively and flower [25]. Periodic burning of fields (traditional cultural method) or herbicide applications allow continued growth of blueberry plants with little interspecific non-*Vaccinium* spp. competition, creating large contiguous mats that characterize commercial fields. Fields are pruned every second year, resulting in a fruit crop in alternate years. Insecticides and fungicides are used to reduce crop loss from insect pests and plant pathogens [26]. In Maine, the bloom lasts for 25–30 days during May and June [27]. There are more than 120 species of bees that have been associated with pollination [28]. In Canada and Maine, native wild bees can contribute up to 28–56% of the set flowers in a field [29]. Honey bees are rented by many growers as a supplemental or “insurance” pollinating field force of bees [30]. Approximately 24% of Maine’s blueberry growers rely solely on wild native bees for their pollination needs, and the rest rely on both wild native bees, honey bees, and/or commercial bumble bees [30]. On a per-bee basis, honey bees are not as efficient a pollinator as many of the native species of bees [30,31,32]. However, the numerical saturation of blueberry fields with honey bees does enhance the fruit set. Both wild native bees and honey bees have been shown to increase the proportion of fruit set as bee density increases in a field in bloom [28,33]. 

The goal of this study was to provide a toxicological baseline for pollinator exposure that can be used as a guide for improving pest management control tactics in the future. The key objective was to determine if trapped honey bee pollen during the bloom had a similar pesticide residue concentration and risk as flowers and the honey bees that were foraging on those flowers in the same fields. In addition, we were interested in seeing if *Osmia* spp. pollen was similar in pesticide residue mixture and concentration to honey bee-trapped pollen collected from the same fields. Lastly, we wanted to determine if honey bees and wild native bees have similar pesticide exposure to one another during the bloom. In other words, do pesticide-contaminated flowers determine risk in a similar way to the pollinators in a blueberry field, or is the risk specific to the taxon of bee foraging in the field?

## 2. Materials and Methods

### 2.1. Study Location

This study was conducted from 2012 to 2014. The sampling of pesticide residues associated with lowbush blueberry fields during the bloom was conducted in the two major lowbush blueberry growing regions in Maine, U.S.A. These geographic areas are referred to as Mid-coast Maine and Down-east Maine, U.S.A. The two regions incorporate Hancock, Knox, Lincoln, Penobscot, Waldo, and Washington counties. Within these counties, blueberry fields were located in many towns, including Addison, Amherst, Appleton, Belfast, Blue Hill, Cherryfield, Columbia, Deblois, Frankfort, Harrington, Hope, Jonesboro, Orland, Sedgewick, Stockton Springs, Union, Warren, Washington, and Wesley. Fields were typically surrounded by a mixed deciduous conifer forest in the Mid-coast region and a spruce-fir forest in the Down-east region. Fields were only sampled in the bloom year. Lowbush blueberry fields are managed in a two-year cropping cycle. The first year is the vegetative growth year and the second year is the bloom and harvest year. All sampled fields in each year were a minimum of 3.1 km from one another, greater than the 1 km average honey bee foraging distance [34] and greater than 2.0 km, the average maximum foraging distance of honey bees [35]. 

### 2.2. Sample Collection

Twelve fields were sampled in 2012, 13 fields in 2013, and 14 fields were sampled in 2014. Of these fields, four individual fields were repeatedly sampled annually for a total of 36 unique fields sampled. Thirty-two of the lowbush blueberry fields were managed with conventional pest management and fertility tactics relying on synthetic pesticides and fertilizers, while four of the fields were managed with organic tactics developed for lowbush blueberry fields [25]. All sampling was conducted during full bloom on the following dates: 2012 (20–26 May); 2013 (31 May–6 June); and 2014 (25 May–12 June). Migratory beekeepers and blueberry growers were contacted for permission to sample honey bee colonies that were brought into wild blueberry fields for pollination during the month-long bloom period (approximately mid-May to mid-June). In each of the 30 fields, migratory honey bee hives were placed by beekeepers at the start of the bloom on wooden pallets with 4–6 hives per pallet. The number of hives per field ranged from 4 to 180, depending upon field size and stocking density (range: 2.5–20 hives/ha). 

Sampling to determine levels of pesticide exposure to honey bees and wild native bees was performed by (1) trapping pollen at honey bee hive entrances (2012–2014), (2) collecting blooming lowbush blueberry flowers (2012 and 2014), (3) capturing honey bee workers foraging on lowbush blueberry flowers (2013 and 2014), (4) capturing native wild bumble bee queens (*Bombus* spp.) on lowbush blueberry flowers (2013); (5) capturing native wild non-*Bombus* bees on lowbush blueberry flowers (2013), and (6) collecting pollen pellets extracted from *Osmia* spp. mason bee trap nest straws (2013). 

### 2.3. Honey Bee-Trapped Pollen Collection

At each field in each year (2012–2014), three random hives were selected from the total aggregation in a field. Pollen was collected during peak bloom using a front entrance pollen trap (Anatomic Front Mount Pollen Trap^®^, Better Bee, Greenwich, NY, USA). Pollen traps were attached to each of the hive entrances without the trapping gate set for 24 h, allowing honey bee foragers to become acquainted with moving through the pollen trap. On the following day, the trapping gate on the pollen trap was closed, and pollen was trapped for 48–72 h [16]. As honey bees with pollen moved through the pollen trap to enter the hive, a proportion of pollen was dislodged from their corbiculae and was collected in the pollen trap bottom tray. Pollen collected from all three pollen traps deployed in a field was pooled. These pooled field-level samples were placed in labeled 250 mL polyethylene cups in the field and taken to the University of Maine Blueberry Hill Research Farm in Jonesboro, ME, for temporary storage in a freezer (−20 °C) until transport to the University of Maine Blueberry Insect Research Laboratory in Orono, ME (see transport, storage, and shipping protocols below). One full 250 mL cup from each field per year was sent for pesticide residue analysis.

### 2.4. Blueberry Flower Collection

In both 2012 and 2014, flowering lowbush blueberry stems were collected in four conventionally managed fields. At each field at peak bloom, 20–30 flowering blueberry stems (average of 70–75 flowers per stem) [25] were cut off at ground level and placed in a 3.8 L Ziploc^®^ freezer bag, labeled, and transported to the laboratory at the University of Maine. In the laboratory, approximately 0.95 L of flowers was separated from stems cut in each field bag and placed in a labeled 0.95 L Ziploc^®^ Freezer Bag (3.8–4.5 g flower samples) and stored in a -80°C freezer (Thermo Scientific^®^, Fisher Scientific, Hampton, NH, USA). 

### 2.5. Osmia spp. Mason Bee Pollen Pellet Collection

In February 2013, 10 wooden block trap nests (3.75 × 13.75 × 20 cm) were nailed to tree trunks at a minimum height of 1.5 m along each of the six southern field edges (six fields sampled in 2013). Trap nest blocks were spaced 2–3 m apart. Each trap nest block had fourteen 6.4 mm diameter holes drilled to a depth of 12.5 cm, and each hole was fit with a tight-fitting paper straw that could be removed for pollen pellet collection (see [36] for more details on trap design and deployment). *Osmia* spp. bees were allowed to colonize the trap nests and provide the straws with pollen pellets and eggs. The most likely *Osmia* species’ pollen pellets (based on the rearing of immatures to adults in the laboratory in 2012 (reviewed in [37]) were *Osmia inspergens* Lovell and Cockerell, *Osmia atriventris* Cresson, *Osmia inermis* Zetterstedt, *Osmia pumila* Cresson, *Osmia tersula* Cockerell. The trap nests were collected toward the end of the bloom (14 June 2013) and taken to the laboratory at the University of Maine in Orono, ME, where the straws were extracted from the blocks, and pollen pellets were dissected from the straws and collected, weighed and placed in labeled vials. Vials were held in a −80 °C freezer (vendor as above). The mean weight of pollen pellets collected per trap nest was 0.45 ± 0.03 g.

### 2.6. Honey Bee Worker Collection

Honey bee workers foraging on lowbush blueberry flowers during peak bloom were collected from 13 fields in 2013 and 14 in 2014. In both years, on sunny days between 10 AM and 3 PM, individual honey bees foraging on blueberry flowers were collected at 6–10 arbitrary locations throughout a field with a snap-cap vial. Bee collecting continued in a field until approximately 30 honey bees were collected. After capturing bees at each location within a field, bees were transferred to a temporary 250 mL vial with a screw cap that was labeled and placed in a cooler containing blue ice packs (Igloo Maxcold^®^ ice blocks, Igloo Co., Katy, TX, USA). The bees were transported immediately back to the laboratory and placed in a -80°C freezer (vendor as above) until they were shipped overnight for pesticide residue analysis. Frozen honey bee samples were weighed prior to analysis. The average sample weight was 2.9 ± 0.1 g.

### 2.7. Native Bumble Bee (Bombus spp.) Queen Collection

In 2013, wild native bumble bee queens (*Bombus* spp.) were hand-collected on blueberry flowers using 100 mL snap-cap vials on sunny days during the hours between 10 AM and 3 PM. One to five queens were collected after visually hunting them from each of 10 fields. Captured queens were held as above for honey bees. The average sample weight was 1.8 ± 0.2 g.

### 2.8. Native Bee (Non-Bombus spp.) Collection

In 2013, wild native bees (non-*Bombus* spp., mostly of the families Andrenidae, Halictidae, Megachilidae, and Colletidae) were hand-collected on sunny days between 10 AM and 3 PM in 13 fields using 100 mL snap-cap vials. Bees (16–20 individuals) foraging on blueberry flowers were collected throughout 10 fields. After capture, bees were handled as above for honey bees. The average sample weight was 0.30 ± 0.05 g.

### 2.9. Shipping of Residue Samples

Samples were shipped overnight express on dry ice to the Connecticut Agricultural Experiment Station for quantitative chemical residue analyses. 

### 2.10. Residue Quantification

Pesticide analyses of samples were conducted at the Connecticut Agricultural Experiment Station, New Haven, CT, U.S.A., which used mass spectrophotometric analysis targeting 140 different pesticides and metabolites after extracting the residue targets using a modified QuECHERs procedure [19,38]. More details of the procedures can be found in [19]. In brief, samples for each site and year (5 g if sufficient material was available or the entire sample if less) were combined with water to a final volume of 15 mL. To each sample, 100 ng of isotopically labeled (d-4) imidacloprid (Cambridge Isotope Laboratories, Andover, MA, USA) was added as an internal standard. Although this does some correction for recovery, it would not account for matrix effects on each individual pesticide (which was cost-prohibitive). The samples were combined with 15 mL of acetonitrile, 6 g magnesium sulfate, 1.5 g sodium acetate, and 150 uL of acetic acid. After shaking and centrifuging, 10 mL of the supernatant was combined with 1.5 g magnesium sulfate, 0.5 g PSA, 0.5 g C-18 silica, and 2 mL toluene. The samples were further shaken and centrifuged. Finally, 6 mL of the supernatant was concentrated to 1 mL for instrumental analysis. 

Residues were analyzed with liquid chromatography/mass spectrometry/mass spectrometry (LC/MS/MS). The LC system was an Agilent 1200 Rapid Resolution system with a Zorbax SB-C18 (Agilent, Santa Clara, CA, USA) Rapid Resolution HT 2.1 × 50 mm, 1.8 µm column using a 3 µL injection with the gradient going from 5% methanol in water to 100% methanol at 0.45 mL/min. In both cases, the LC was coupled to a Thermo-LTQ (Thermo-Fisher, Waltham, MA, USA) linear ion trap mass spectrometer. The system was operated in the positive ion electrospray mode, with a unique scan function for each compound, allowing for MS/MS monitoring. Detection limits varied with the amount of sample available but were in the single ng pesticide/g pollen level (commonly abbreviated as ppb (parts per billion)) or lower for pesticides reported herein. If a pesticide was not detected, it was reported as such, not as a concentration of 0 ppb. 

For enhanced specificity in pesticide detection at very low concentrations, we chose to use only our available LC/MS-MS. While providing greater confidence in our data on pesticides detected during a multi-residue screen, this choice precluded the detection of some classes of pesticides (such as pyrethroid insecticides. Also, we could not detect chlorothalonil, only its hydroxyl metabolite), which can require additional sample cleanup steps and GC/MS in electron impact or negative chemical ionization modes. Limits of detection (LOD) ranged from 0.5 to 30 ppb depending upon the matrix from which the residues were extracted (most compounds had an LOD of <5 ppb). Residue detection limits for most compounds were much less than an order of magnitude difference across all matrices (flowers, trapped pollen, *Osmia* spp. pollen, honey bees, bumble bees, and other native bees) analyzed. Therefore, we felt that the statistical analysis and conclusions across matrices were legitimate without adjusting the pesticide detection profiles (see [39] for an adjustment methodology).

### 2.11. Risk Quotient Estimation

Honey bee Risk Quotients (RQ) were calculated using the oral adult honey bee LD_50_ for specific pesticides and metabolites and an adult honey bee pollen consumption rate of 9.5 mg/day (see [40]). When the oral adult honey bee LD_50_ was not available, the contact LD_50_ was used instead. We are aware that a “Honey Bee Risk Quotient” may only provide a relative risk measure for the honey bee, but because toxicological risk information is lacking for most other bee species, we decided to use the “Honey Bee Risk Quotient” as a proxy for wild native pollinators in Maine wild blueberry. The investigators, de Assis et al. (2022) [41], showed that while not precise, a “Honey Bee Risk Quotient” can be used to make a relative comparison of pesticide risk among honey bees and wild native bees.

### 2.12. Statistics 

All statistical analyses were conducted using the statistical software JMP Pro, version 16 [42]. Four groups of analyses were conducted. In each group, data used were samples that were collected in the same fields at the same period. The analyses were as follows: (1) pesticide residues in lowbush blueberry flowers compared to pesticide residues in honey bee-trapped pollen (four fields in 2012, 4 fields in 2014); (2) pesticide residues in/on captured honey bee foragers compared to pesticide residues in honey bee-trapped pollen (seven fields in 2013, 13 fields in 2014); (3) pesticide residues in *Osmia* spp. pollen pellets compared to pesticide residues in honey bee-trapped pollen (six fields in 2013); and (4) pesticide residues in/on captured honey bee foragers compared to pesticide residues in foraging *Bombus* spp. queens, and pesticide residues in foraging non-*Bombus* wild native bees (ten fields in 2013). Dependent variables selected for a model in each group were: (1) the number of unique pesticide residue compounds detected in the matrix (square root transformed); (2) the total concentration of pesticide residues detected in the matrix (logarithm transformed); (3) Risk Quotient for each matrix. Models for each dependent variable within a group were general linear models with the matrix as a categorial treatment independent variable and the field site as a statistical block variable. Evaluation of the final general linear model with the assessment of a lack of fit F-test and correlation among estimates of fixed effects. Also, studentized residuals and their Bonferroni 95% simultaneous confidence limits were used to identify potential outliers and homo-scedasticity. The normality of residuals was tested with the Shapiro–Wilk test.

In addition to the general linear models constructed to assess differences in pesticide residue measures among matrices, a linear correlation was conducted in order to assess the degree of association among the pesticide residue mixtures detected in the matrices and the incidence or frequency (proportion of field sites found) of occurrence of each detected residue among the matrices. Each datum was the frequency of a specific detected residue compound in one matrix paired with the frequency of the same detected residue compound in another matrix. A strong, significantly positive correlation indicates a high similarity of the mixtures of pesticide residues among the two matrices. 

## 3. Results

### 3.1. Honey Bee-Trapped Pollen Compared to Flowers

**Figure 1:**
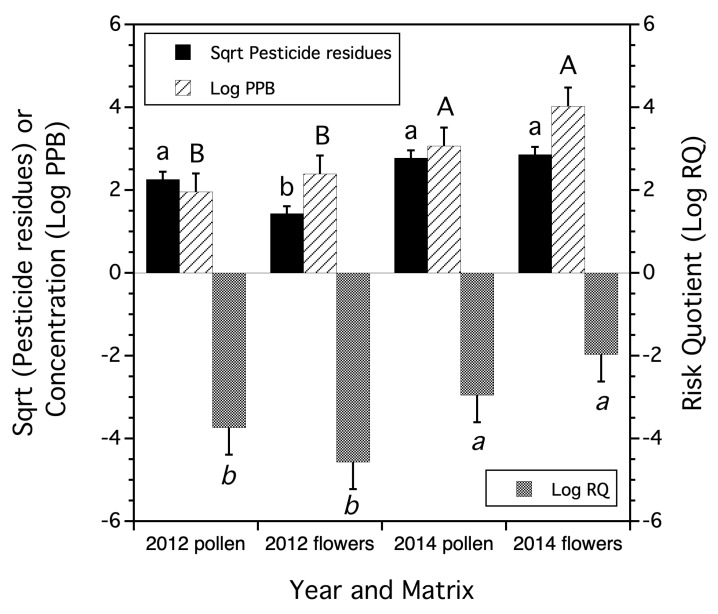
The mean number of pesticide residues (square root transformed), mean logarithm transformed concentration of residues, and mean logarithm Risk Quotient found in trapped honey bee pollen versus blueberry flowers collected in 2012 and 2014. Means and standard errors are the least square estimates from a general linear model. Bars of the same residue measure followed by the same letters are not significantly different from one another (Tukey HSD test, α = 0.05). Lower Risk Quotient values suggest less risk to honey bee colonies.

An interaction for the number of pesticide residues detected (square root transformed) between year and matrix (trapped honey bee pollen vs. blueberry flowers) was found for the 2012 and 2014 trapped pollen and flower collections (*F*_(1,12)_ = 6.393, *p* = 0.027, Figure 1). Differences in total concentration (log ppb) and Risk Quotient (log RQ) were found between years pooled over trapped pollen and flowers, but there was no interaction between year and the matrix (pollen or flowers) (*p* > 0.05, Figure 1). 

There was an overlap between the pesticide residue compounds found on flowers and those found in trapped honey bee pollen. Over both years (2012 and 2014), 12 residue compounds were found on flowers (*n* = 8 sites), and 17 residue compounds were detected in the trapped honey bee pollen (*n* = 8 sites). The number of insecticides and herbicide residues detected on flowers and in trapped pollen were the same (*n* = 5 insecticides, *n* = 2 herbicides). Trapped pollen had more fungicides detected (*n* = 7) compared to flowers (*n* = 4). There were three acaricide residues, presumably a result of Varroa mite (*Varroa destructor* Anderson & Trueman) control, which were detected in trapped honey bee pollen (83% of the eight field sites having at least one acaracide residue). None of these acaracide residues were detected on flowers at any of the paired collection field sites. There were six compounds (axoxystrobin, boscalid, chlorothalonil, propiconazole, hexazinone, and phosmet) that were found in both types of samples (flowers and trapped honey bee pollen) in the eight paired field sites over the two years. The frequencies of field sites where common compounds were found on flowers compared to the frequency of sites with these same compounds in trapped pollen were highly correlated (*r* = 0.822, *p* = 0.044), suggesting that much of the source of pesticide residues found in trapped pollen originated from flowers in the lowbush blueberry fields. Even though no differences in residue concentrations (PPB) were found among trapped pollen and flowers there was a non-significant trend (*p* = 0.122) for higher concentrations in flowers compared to trapped pollen. 

### 3.2. Honey Bee-Trapped Pollen Compared to Honey Bee Workers

**Figure 2:**
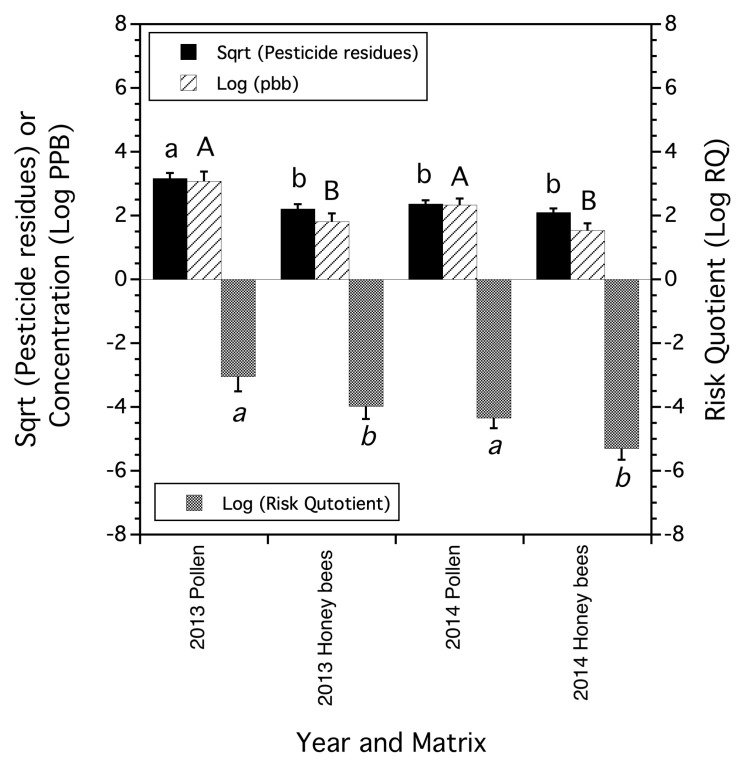
The mean number of pesticide residues (square root transformed), mean logarithm transformed concentration of residues, and mean logarithm Risk Quotient found in trapped honey bee pollen versus honeybee workers collected in 2013 and 2014. Means and standard errors are the least square estimates from a mixed model. Bars of the same residue measure followed by the same letters are not significantly different from one another (Tukey HSD test, α = 0.05). Lower Risk Quotient values suggest less risk to honey bee colonies.

In the two years (2013 and 2014) that trapped pollen and honey bee workers were collected in the same 20 fields (*n* = 7 in 2013, *n* = 13 in 2014), there was evidence for a matrix (honeybees vs. trapped pollen) × year interaction (*F*_(1,17)_ = 14.001, *p* = 0.002) when the number of residue types was the dependent variable (square root transformed number of residues). Honey bee-trapped pollen in 2013 contained more types of pesticide residue compounds than trapped pollen in 2014 and more types of residue compounds than honey bee workers in 2013 and 2014, which were not different from the 2014 trapped pollen (Figure 2). The total concentration of pesticide residues (log PPB) in honey bee-trapped pollen and worker honey bees showed evidence of a matrix main effect (*F*_(1,17)_ = 26.614, *p* < 0.0001). In both years, total residue concentration was higher in trapped pollen than in honey bee workers (Figure 2). The same pattern that was observed for total residue concentration was observed with the honey bee Risk Quotient (*F*_(1,17)_ = 12.859, *p* = 0.002), where in both years, trapped pollen had a higher risk than the risk found in honeybee workers (Figure 2).

Over both years, 11 pesticide residue compounds were detected in trapped pollen, and 10 residue compounds were detected on honey bee workers. In the trapped pollen, six fungicides, four herbicides, one insecticide, and two acaricides (Varroacides) were detected. On worker bees, no insecticide residues were detected, but four fungicide residues, three herbicide residues, and one acaracide residue were detected. Of these detected compounds, seven were in common among the two matrices (chlorothalonil, propiconazole, boscalid, axoxystrobin, hexazinone, and two residues of the Varroacide, amitraz). A significant correlation among the paired matrices and their frequencies across fields sampled suggested that of those compounds detected in common for both matrices, they occurred in a similar intensity for trapped pollen and worker bees (*r* = 0.775, *p* = 0.043). 

### 3.3. Honey Bee-Trapped Pollen Compared to Osmia spp. Pollen

**Figure 3:**
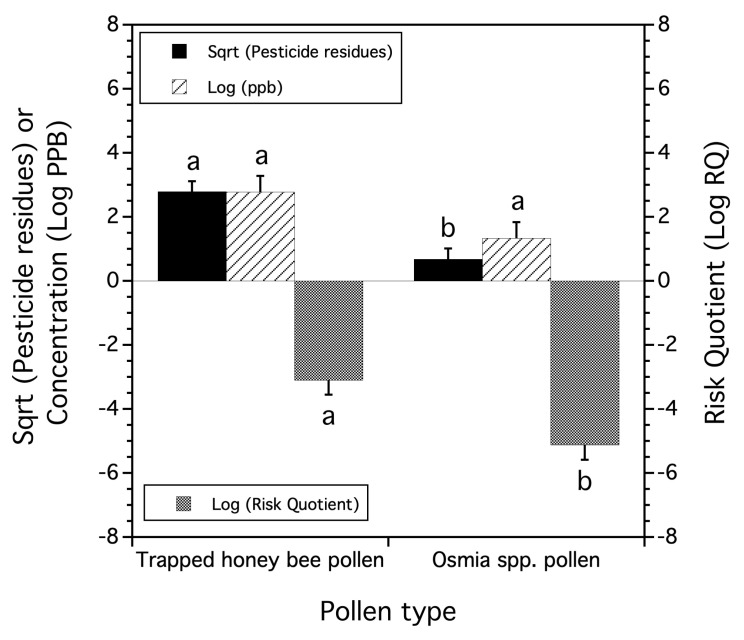
The mean number of pesticide residues (square root transformed), mean logarithm transformed concentration of residues, and mean logarithm Risk Quotient found in trapped honey bee pollen versus *Osmia* spp. pollen collected in 2013. Means and standard errors are the least square estimates from a mixed model. Bars of the same residue measure followed by the same letters are not significantly different from one another (Tukey HSD test, α = 0.05). Lower Risk Quotient values suggest less risk to honey bee colonies.

In the 2013 sampling of six lowbush blueberry fields, we found that there were significantly more pesticide residue types in honey bee-trapped pollen from lowbush blueberry fields than the *Osmia* spp. pollen collected from field edge wooden trap nests in the same fields (*F*_(1,5)_ = 15.154, *p* = 0.012, Figure 3). There were 19 total pesticide residues detected in the trapped pollen from the six paired blueberry fields, but only seven total pesticide residues were found in the *Osmia* spp. pollen collected from the same six fields. All seven of the pesticide residues found in *Osmia* spp. pollen were also found in honey bee-trapped pollen. When the proportion of fields containing these seven common compounds was compared between trapped pollen and *Osmia* spp. pollen, a significant linear correlation was found to exist (*r* = 0.964, *p* = 0.0005). Therefore, we can hypothesize that a significant proportion of the variation in pesticide residues found in trapped pollen is similar to the residue composition found in *Osmia* spp. in the paired lowbush blueberry fields. *Osmia* spp. pollen contained only fungicide (*n* = 3) and herbicide (*n* = 4) residues, whereas trapped honey bee pollen contained all four pesticide use classes (fungicides (*n* = 7), herbicides (*n* = 7), insecticides (*n* = 3), and Varroacides, i.e., acaracides (*n* = 2)). However, the average total pesticide residue concentration of trapped honey bee pollen was not significantly different from the collected *Osmia* spp. pollen (*F*_(1,5)_ = 3.447, *p* = 0.123, Figure 3). The non-significant trend observed was toward a greater pesticide residue concentration in trapped honey bee pollen compared to *Osmia* spp. pollen. However, risk, as measured by the log of the Risk Quotient (RQ calculated from both pollens as the risk to honey bee workers), was significantly greater in trapped honey bee pollen than the *Osmia* spp. pollen (*F*_(1,5)_ = 7.405, *p* = 0.042, Figure 3). Therefore, trapped honey bee pollen contained significantly more residue types and a higher RQ when compared to *Osmia* spp. pollen.

### 3.4. Comparisons of Pesticide Residues Found on Three Bee Taxa

In 2013, three taxa of bees (honeybee workers, wild native bumble bee queens, and wild native non-bumble bees) were collected from 10 lowbush blueberry fields sampled during the bloom.

**Figure 4:**
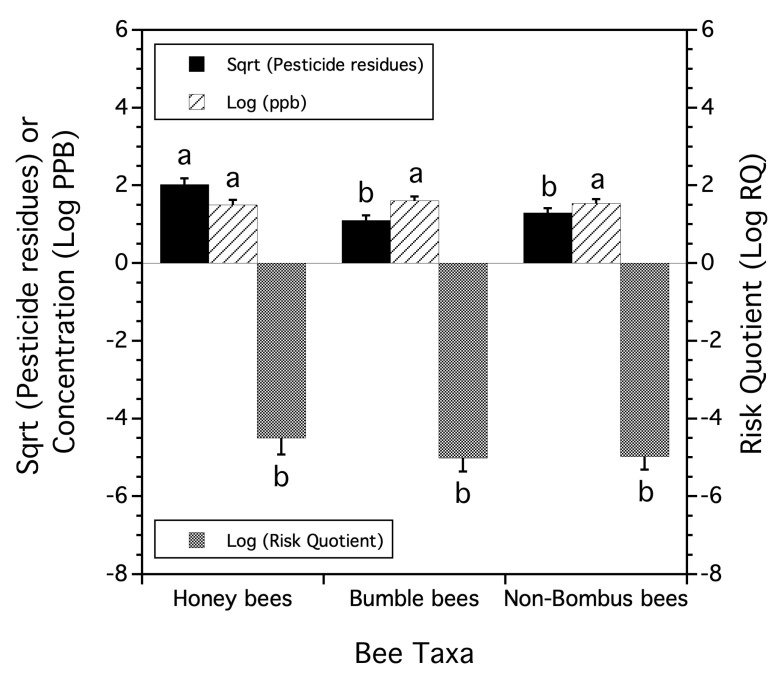
The mean number of pesticide residues (square root transformed), mean logarithm transformed concentration of residues, and mean logarithm Risk Quotient found in three bee taxa collected in 2013. Means and standard errors are the least square estimates from a mixed model. Bars of the same residue measure followed by the same letters are not significantly different from one another (Tukey HSD test, α = 0.05). Lower Risk Quotient values suggest less risk to honey bee colonies.

Honey bee workers had significantly more types of pesticide residue compounds (ca. 3X) on/in their bodies than bumble bee queens or non-*Bombus* native bees (*F*_(2,14)_ = 15.766, *p* = 0.007, Figure 4). There were no differences in total residue concentrations (Log PPB) among the three bee taxa (*p* = 0.854, Figure 4), nor the Risk Quotients (Log RQ) among these three taxa (*p* = 0.649, Figure 4). 

When summing over all ten field sites, ten pesticide residue compounds were detected on honey bees compared to four residues on bumble bees and six residues on non-Bombus native bees. Four of the pesticide residues were common to all three bee taxa. These residues were the fungicides axoxystrobin, chlorothalonil, propiconazole, and the herbicide hexazinone. Only one insecticide was detected and it was from a single site on a bumble bee sample. Acaracides (*n* = 3 residue types) were only found on honey bees, suggesting that the bees were exposed to these in the hive as a result of beekeeper treatments for control of *Varroa* mite. The only bee taxa where the residue frequencies of site occurrences were correlated was the association among bumble bees and non-*Bombus* native bees (*r* = 0.943, *p* = 0.049). This might suggest that bumble bee queens and non-*Bombus* native bees foraged in a similar manner but differently from honey bee workers, such that potential exposure differed in these paired field sites. 

## 4. Discussion

In general, we found that specific pesticide residues detected on flowers were highly correlated with the same pesticide residues detected on trapped honey bee pollen. Fewer types of residue compounds were found on flowers than trapped pollen, but overall total residue concentrations were not significantly different between flowers and trapped pollen. Honey bee risk quotients based upon residues on flowers and trapped pollen were not significantly different. This may be because an equal number of insecticide residues (compounds with the highest RQ values) were found on flowers and trapped pollen. Overall, the comparison of residues on flowers to trapped pollen suggests that the honey bee colonies placed in the fields where the flowers were sampled were primarily foraging in those fields. For studies of honey bees foraging on sampled crop plants [43,44,45], high correlations between residues in bee-collected pollen and residues detected in nectar and residues have been reported. It is generally assumed that crop plants in the field are those plants that honey bees forage on and the pollens that comprise trapped pollen collections. However, this does not always appear to be the case. Alburaki et al. (2018) [46] demonstrated that non-crop pollen comprised the majority of trapped honey bee pollen in agricultural landscapes in Tennessee (USA), but Raimets et al. (2020) [47] showed that pesticide residue levels in various honey bee hive matrices were independent of the proportion of non-crop pollen in trapped pollen. 

Few studies report sampling of both crop plant matrices and trapped pollen from the same fields simultaneously. Analysis of samples of highbush blueberry flowers (*Vaccinium corymbosum* L.) and honey bee-trapped pollen in Michigan (USA) showed that residues found on flowers were not similar to the residues found in trapped pollen. The results led to the conclusion that most of the pesticide risk to honey bees was due to “off-farm” sources [48]. This is quite different than what we found in Maine lowbush blueberry [49]. Many Maine blueberry fields are embedded in Acadian or Boreal Forest landscapes and so bee foraging outside of lowbush blueberry fields in adjacent floral landscapes may not be common [50]. In support of this conjecture, corbicula pollen load analysis of honey bees collected in forest-embedded lowbush blueberry fields showed that 87% of the pollen was lowbush blueberry pollen [51]. Large aggregations of contiguous lowbush blueberry fields in Maine do exist, and honey bee colonies placed in blueberry fields with neighboring adjacent blueberry fields may forage out of their immediate “home” field. However, this may be dependent upon nectar and pollen availability within the field and the size of the field [52]. The field sites of ours where the types of pesticide residues were not identical between flowers and trapped pollen might reflect bees foraging out of the “home” field where both colonies were placed, and flowers sampled [50]. 

Not surprisingly, pesticide residues in honey bee-trapped pollen reflected the pesticide residue exposure that we found in field-captured foraging honey bee workers. Approximately 70% of the compounds found on trapped pollen were also detected in honey bee foragers. Insecticides were not found on captured honey bees, while one insecticide was found in trapped pollen. Fungicides, herbicides, and Varroacides were found on both trapped pollen and captured foraging workers. In both years of sampling (2013 and 2014), significantly greater concentrations of pesticide residues were found in trapped pollen compared to honey bee foragers, and the same was found for the honey bee Risk Quotient. Therefore, it appears that pesticide residues are concentrated in trapped pollen relative to the foragers that collect the pollen. The number of types of pesticide residues contributing to exposure can best be explained as an interaction over time. In 2013, trapped pollen contained significantly more pesticide residue compounds than foragers, while in 2014, the number of pesticide residue compounds was not different between trapped pollen and foragers. The higher concentrations and Risk Quotients of trapped pollen relative to captured honey bee foragers have also been documented by Kasiotis et al. [53]. They found that trapped pollen had concentrations ranging from approximately 1- to 2-orders of magnitude higher than honey bee workers. The authors did not speculate on why this apparent concentration of residues occurs in pollen relative to foragers. We suggest that this phenomenon might be due in part to a foraging time bias. Captured workers represent bees that have not finished their foraging bout, and the samples of bees that we collected were, on average, halfway through their foraging trip. Trapped pollen is the result of a finished successful foraging bout. Therefore, the time that was accrued from a bee leaving the hive to commence foraging and, thus, the amount of time in which exposure occurred was greater for the trapped pollen loads compared to the captured bee forager samples. Another source of the difference in residue concentrations in trapped pollen compared to foragers is that honey bees often alight on blueberry stems or leaves to groom pollen off of their bodies onto the corbiculae (author’s observations, FAD), and thus, residues may be transferred from the waxy cuticle of workers onto the pollen loads. While transferring pollen onto the corbiculae, concentrated nectar from the bee’s crop, which is obtained in the hive prior to each foraging bout, is added to the pollen, providing adhesion among pollen tetrads [54].

Do native bees foraging in lowbush blueberry fields have greater exposure to pesticide residues than honey bees? To answer this question, first, we compared the honey bee-trapped pollen to *Osmia* spp. pollen. *Osmia* mason bees are a diverse assemblage of bee pollinators in Maine lowbush blueberry fields, and 16 species have been recorded [38,55]. We found that trapped pollen collected from hives deployed in the same lowbush blueberry fields as *Osmia* spp. Trap nest pollen had significantly more pesticide residue types than *Osmia* pollen, although seven residues were correlated among the matrices when the frequency of site occurrence was used as a measure of residue incidence. However, residue concentration was not different between pollen types. The calculated “Honey Bee Risk Quotient” applied to both honey bee-trapped pollen and *Osmia* spp. pollen was significantly greater for honey bee-trapped pollen. This suggests that *Osmia* spp. bees may have a lower exposure rate than honey bees when considering the number of pesticide residue compounds, no difference when the total concentration of all residues was considered, and if the “Honey Bee Risk Quotient” can be viewed as a proxy for *Osmia* spp. bees, then the overall risk to them appears to be significantly less in lowbush blueberry fields than for honey bees. Laboratory toxicological topical bioassays of *Osmia cornifrons* Radoszkowski and *A. mellifera* revealed that the honey bee is not a good proxy for estimates of risk from select pesticides [56]. However, other bioassays that compared acute effects between *Bombus terrestris audax Harris, Osmia bicornis* (L.), and *A. mellifera* found that there were significant interspecific differences that varied through time, but overall, the magnitude of these differences (in terms of treatment effect ratios) was generally comparable. This led the authors to conclude that honey bees might be used as a wild bee proxy if an assessment factor to account for interspecific variation was used in calculations of risk [57]. Because of the uncertainty concerning the *Osmia* species’ composition that comprised the pollen samples we collected, it is not possible to know if the “Honey Bee Risk Quotient” is realistic to apply to the *Osmia* spp. community foraging in Maine lowbush blueberry fields. Currently, only a few pesticides have been evaluated for acute toxicity to *Osmia* spp. adults, and even fewer for larvae. Therefore, the best measures of exposure are the number of pesticide compounds and the total concentrations of pesticide residues detected in their pollen.

Assessment of exposure to residues found associated with adult foraging honey bees, queen bumble bees, and non-*Bombus* wild native bee species all collected in the same lowbush blueberry fields at the same time suggested that only the number of types of pesticide residues differed. Significantly, more pesticide residue compounds were detected on honey bee foragers than on bumble bee queens or non-*Bombus* native bees. The total pesticide residue concentrations on the three adult bee taxa groups were not different. Also, the proxy “Honey Bee Risk Quotient” was not different if applied to the three taxa groups. Three fungicides and one herbicide were common to all three groups, while Varroacide residues (*n* = 3 compounds) were only found on honey bee foragers. Several studies have estimated the pesticide exposure of wild native bees in crop fields [58,59,60,61], and others have shown that exposure to pesticide residues in agricultural landscapes may reduce the abundance and species richness [12], growth rate and reproduction [14], and disease susceptibility [62]. 

Several studies have compared pesticide residue exposure in wild native bees to honey bees. Our sampling in Maine lowbush blueberry fields found that honey bees had significantly more types of pesticide residue compounds associated with their bodies than bumble bee queens or non-*Bombus* wild native bees. The total concentrations of pesticide residues and the “Honey Bee Risk Quotients” applied to all three taxa groups did not differ. This is somewhat in contrast to other findings [63], where wild native bees, compared to honey bees, were exposed to similar numbers of pesticide residues in a California agricultural landscape. Although similar to our study, they found that the total concentrations of pesticide residues were not different among honey bees and wild native bees. Our study also had similar findings to theirs in that pesticide mixtures varied by bee type. Another study [63] also found that as wild bee body size increased, the number of contaminant pesticide residues increased. Because we did not dissect the wild native bee community beyond two large taxa groups, *Bombus* spp. and non-*Bombus* native bees, we are not able to determine species-specific differences in exposure to pesticide residues. Using expert opinion, a study [64] evaluated bee traits across several taxa that might make bees vulnerable to pesticide exposure. The authors hypothesized that wild native bees might be more vulnerable to pesticide residues than honey bees due to traits that determine the degree of exposure and the ability to recover from reductions in abundance and that differences between wild native bee species might exist.

## 5. Conclusions

A general finding in our study was that flower pesticide residues reflected the pesticide contamination of honey bee-trapped pollen and honey bee workers. Honey bees sometimes had a higher exposure level than wild bees, their pollen, or blueberry flowers. Honey bee exposure was not consistently higher than wild bees, but wild bees never had higher exposures than honey bees. A summary follows for the three measures of exposure: number of residue compounds detected; total concentration of residues; and a measure of risk, estimated as the honey bee RQ. Honey bees, compared to their trapped pollen, had lower exposure as measured by the number of compounds detected in one (2014) of two years. However, in both years (2013 and 2014), honey bees had lower exposure than their trapped pollen in the average total pesticide residue concentration and the average RQs. 

Lowbush blueberry flowers had lower numbers of pesticide compounds detected in one year (2012) than honey bee-trapped pollen, but in the other year, 2014, flowers and trapped pollen were not different in the average number of compounds detected. The total residue concentrations and the RQs were not different between flowers and honey bee-trapped pollen in either of the two years. In both years, honey bees had average exposure levels that were lower than trapped pollen when measuring total pesticide residue concentration and RQ. 

We found that honey bee-trapped pollen compared to *Osmia* spp. pollen contained significantly higher numbers of pesticide residue compounds and higher average RQs. There were no differences in the average total pesticide residue concentration between these two pollen types. There were only differences between the three bee taxa in the average number of pesticide residues detected and not the average total concentration of pesticide residues or average RQs. Honey bees had a significantly higher average number of pesticide residues detected on their bodies compared to bumble bee queens and non-*Bombus* wild native bees, which were not different from one another in the detected number of compound residues. 

Varroacides were detected in honey bee-trapped pollen and on captured honey bee foragers. Varroacides were missing from flowers, *Osmia* spp. pollen, bumble bee queens, and non-*Bombus* wild native bees. The honey bee colonies used for pollination of lowbush blueberry were almost entirely migratory colonies that were moved to Maine from the southern and western U.S. [30]. These colonies are often exposed to pesticide residues during pollination from almonds and other early spring crops prior to their deployment in lowbush blueberry fields in Maine [65]. In addition, Varroacides are often applied to colonies either just before arriving in Maine or during the start of lowbush blueberry pollination. Therefore, contamination of trapped pollen with Varroacides is most likely picked up by foragers in the hive and then transferred to pollen in the field by bodily contact or nectar regurgitation on pollen during the collection or grooming of pollen from the honey bee’s body to the corbiculae. It seems reasonable that the same might occur with other pesticide residues in the hive that are not common pesticide residues that wild native bees foraging on lowbush blueberry will encounter as a result of pesticide applications by growers.

It is also possible that differences in foraging pattern and distance might determine the number of pesticide residues that are picked up by honey bees compared to wild native bees. Honey bees are known to forage over larger territories than wild native bees [66,67]. In addition, within blueberry fields, it has been observed that honey bees tend to visit fewer flowers per clone (a genetically unique plant) and more clones during a foraging bout than bumble bee queens, *Andrena* spp., and *Osmia* spp. bees [31]. In addition, many wild native bees nest on field edges, except for several species of Andrenide [68]. Field edges may not receive the pesticide coverage that field interiors receive, thus resulting in bee species that forage close to their nest near field edges having lower exposure to pesticide residues. Therefore, we speculate that more dispersed foraging by honey bees than wild native bees might expose them to a greater number of pesticide residue compounds.

## Data Availability

Data will be shared upon request.
